# Investigating food-related behaviors in Smith-Magenis syndrome: tailoring a questionnaire for a rare disease

**DOI:** 10.1007/s44162-026-00193-3

**Published:** 2026-04-29

**Authors:** Citrine Elatrash, Theresa A. Wilson, Alexis C. Wood, Sarah H. Elsea, Stephanie Sisley

**Affiliations:** 1 Department of Pediatrics, USDA/ARS Children’s Nutrition Research Center, Baylor College of Medicine, 1100 Bates St., Houston, TX 77030 USA; 2 Department of Molecular and Human Genetics, Baylor College of Medicine, Houston, TX 77030 USA; 3 BCM-Human Genome Sequencing Center, Baylor College of Medicine, Houston, TX 77030 USA

**Keywords:** Smith-Magenis syndrome, Food-related behaviors, Obesity, Questionnaire development, Hyperphagia

## Abstract

**Purpose:**

Accurate measurement is essential for tracking changes in clinical outcomes. Individuals with Smith-Magenis syndrome (SMS) exhibit challenging and unique food-related behaviors. We sought to determine the best tool to capture their unique food-related behaviors.

**Methods:**

We conducted focus groups with caregivers of individuals with SMS to evaluate two commonly used questionnaires for food-related behaviors– the Food Related Problems Questionnaire (FRPQ) and the Hyperphagia Questionnaire for Clinical Trials (HQ-CT). Based on caregiver input and clinical expertise, we adapted these existing measures into a new tool: the SMS-FRPQ. We then validated this instrument for internal consistency and concurrent validity using online responses from 125 caregivers.

**Results:**

Caregivers (*n =* 24) indicated neither the FRPQ or HQ-CT fully captured their child’s food-related behaviors; however, the newly developed SMS-FRPQ was deemed comprehensive by a new group of caregivers (*n =* 19). The SMS-FRPQ demonstrated strong internal consistency, with a Cronbach’s alpha of α = 0.87 [0.84–0.90]. Factor analysis indicated a three-factor model was a good fit (comparative fit index (CFI) = 0.9, Tucker-Lewis index (TLI) = 0.88). The three factors each showed good internal consistency: Desire for Food, α = 0.80 [0.74–0.85]; Takes Food, α = 0.87 [0.83–0.90]; Satiety Impairment, α = 0.83 [0.77–0.87]. The SMS-FRPQ showed higher internal reliability than the FRPQ. Concurrent validity was supported through alignment with similar items from the HQ-CT or Behavioral Problems Inventory (BPI-01).

**Conclusions:**

The SMS-FRPQ has 14 items across 3 different factors and 5 additional clinically relevant items. This validated tool may be useful for tracking food-related behavior outcomes in clinical trials for this high-risk population.

**Supplementary Information:**

The online version contains supplementary material available at 10.1007/s44162-026-00193-3.

## Introduction

Smith-Magenis syndrome (SMS) is a complex neurobehavioral disorder caused by either a deletion of chromosome 17p11.2 or a pathogenic variant in *RAI1* [19, 36]. The syndrome includes failure to thrive, hypotonia, speech and motor delays, intellectual disability, hearing loss, vision impairment, sleep disturbance, obesity, and behavioral abnormalities like self-injurious behaviors, aggression, temper tantrums, and stereotypical behaviors [11, 19]. The incidence of SMS is estimated to be between 1:15,000 to 1:25,000, which calculates to less than 20,000 people with the disease in the United States, clearly meeting the definition of a rare disease.

Obesity has been reported in approximately one-third to one-half of all SMS patients, with higher prevalence in persons > 9 years of age [10, 17]. Pathogenic variants in the *RAI1* gene predispose individuals to having obesity more than those with deletions of chromosome 17p11.2 [17]. Recent studies suggest that rare variants in *RAI1* that contribute to obesity are likely underdiagnosed [1, 8, 46]. Interestingly, there may also be sex-specific effects of SMS, as in analysis of all previously published cases, almost 70% of females were reported to exhibit eating/appetite problems compared to only 21% of males [17]. Further supporting the genetic-driven obesity are mouse studies that show either deletion of the 17p11.2 locus (including *Rai1* on chromosome 11 in mice) or haploinsufficiency of *Rai1* alone results in increased body weight and fat mass [10, 28]. It is well known that abdominal obesity, which is a prevalent type in the SMS population and in mouse models [3, 10, 37], increases the risk of obesity-related comorbidities such as hypertension, diabetes, and metabolic syndrome [32]. Thus, it is not surprising that there is an increased prevalence of hypercholesterolemia in individuals with SMS [37] and abnormal glucose tolerance in *Rai1* haploinsufficient mice [28, 37]. Given all of these findings, effective treatments for obesity and prevention of obesity in SMS are urgently needed.

While low satiety leading to excessive intake would appear to be a reasonable hypothesis for the development of obesity in SMS, there is discrepancy regarding the magnitude of abnormal satiety in the SMS population. Mice with haploinsufficiency of *Rai1* have increased food intake and impaired satiety [10] but not necessarily excessively sufficient to support their weight gain. However, after a fast, *Rai1*^±^ mice overeat with sustained food intake over the course of the next 12–48 h. This is in contrast to wildtype mice which overeat for the first 12 h and then have decreased food intake for the next 36 h [10]. Additionally, *Rai1*^±^ mice have lower expression of *Pomc* and *Bdnf* in the hypothalamus, which are both known to have important appetite-suppressing effects [10]. On the other hand, stimulants, well known to have appetite-suppression as a side effect, are reportedly ineffective for weight control in individuals with SMS [5]. Additionally, a recent clinical trial showed no change in BMI after short term (three months) treatment with setmelanotide, a melanocortin-4 receptor agonist [29]. This would imply that individuals with SMS do not suffer only from a lack of satiety, per se, but perhaps have other features driving their obesity.

Indeed, our published data support that food-related behaviors are complex in SMS and relate not only to hunger but also to an individual’s need for autonomy and justice [18]. Some of their food-related behaviors stemmed from underlying aspects of their biology due to Smith-Magenis syndrome, including their significant behavioral issues [2, 19]. We have previously published that self-injury, yelling/screaming, and aggressive/destructive behavior are very common in the SMS patient population [2]. Not only are these behaviors common, but they are more common in both frequency and severity in the SMS population than a comparison group of adults with intellectual disability [2]. Thus, we found that frustration with food also resulted in aggressive behaviors [18], which were likely not related to hunger per se. Additionally, individuals with SMS craved autonomy, fairness, and ownership over food, even to the point of taking/hoarding food without eating it [18]. Again, these demonstrate food-related behaviors may stem from both hunger and non-hunger needs.

While published data support overall similarities between PWS and SMS [2], critical assessment of our published and unpublished data, however, has revealed a striking discrepancy between satiety scores and other items on available food-related questionnaires. Caregivers of children and adults with SMS score “impaired satiety” similarly to reports of others with intellectual delay; however, 57% of caregivers reported locking away food. Thus, if individuals with SMS do not have significantly impaired satiety as the HQ-CT would suggest, there should not be such a high need for food to be locked away. Further, if food is locked away or access to food is otherwise limited, then answers to the questions on standard satiety/hunger-related questionnaires related to eating at night and food-seeking/sneaking may be skewed, resulting in inaccurate or misleading satiety scores. We hypothesized that a syndrome-specific questionnaire for food-related behaviors was needed for the SMS population.

In this project, we used qualitative methods to understand caregiver experiences with currently available food-related behavior questionnaires, combined with our previously published data understanding food-related behaviors in individuals with SMS [18] and tailored a new questionnaire for the SMS population (Smith-Magenis Syndrome-Food-Related Problems Questionnaire; SMS-FRPQ). We show here the validation of this tailored questionnaire in the SMS population.

## Methods

This study was approved by the Baylor College of Medicine Institutional Review Board and was performed in accordance with the ethical standards of the 1964 Declaration of Helsinki and its later amendments.

### Recruitment, consent, and participants

We recruited caregivers of individuals with SMS from the 2022 and 2024 International Parents and Researchers Interested in Smith-Magenis Syndrome (PRISMS) Research Conferences, the SMS Patient Registry (a world-wide registry funded by PRISMS and administrated by Baylor College of Medicine), as well as the PRISMS Facebook group. For focus groups, verbal consent was obtained. For online questionnaire completion, caregivers were instructed in writing that completion of questionnaires implied consent. No written informed consent was obtained.

### Development of SMS-FRPQ (see Fig. 1)

**Fig. 1:**
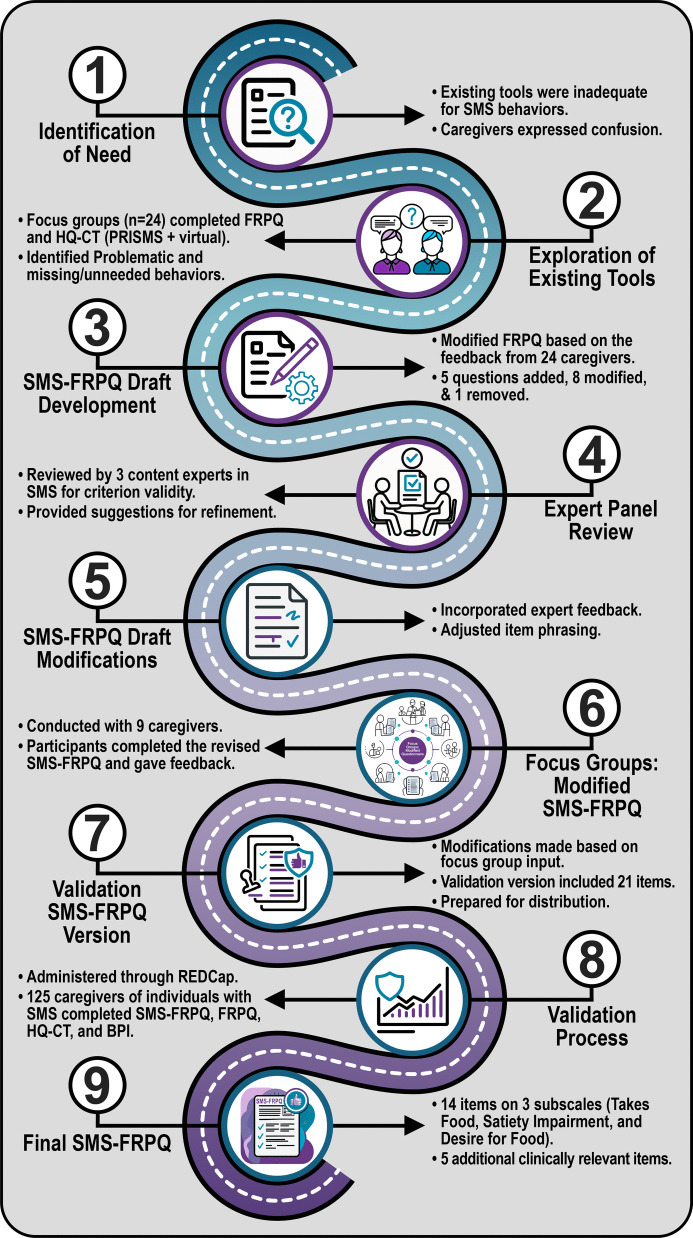
SMS-FRPQ Development Process. The development of the SMS-FRPQ began with (1) identifying the need for a new questionnaire and then (2) exploring the difficulties with existing tools. (3) A modified version of the FRPQ, named the SMS-FRPQ, was developed with caregivers and underwent (4) expert panel review. (5) Modifications were made to the SMS-FRPQ draft and then it was verified by (6) a second round of caregiver focus groups and modifications were made, resulting in the (7) validation version of the SMS-FRPQ. The validation version was completed by 125 caregivers and underwent (8) validation analysis, resulting in a (9) final SMS-FRPQ.

Qualitative work resulting from this study regarding food-related behaviors in the SMS population was previously published [18]. However, during these focus groups, caregivers (*n =* 24) were also asked to complete the Food Related Problem Questionnaire (FRPQ) [34] and the Hyperphagia Questionnaire for Clinical Trials (HQ-CT) [12]. Using semi-structured interview questions, groups discussed the behaviors not addressed on the questionnaires and which questions were difficult to answer based on their experience. Based on this work, we determined to create an instrument to encompass the most prevalent food-related behaviors. The research team created a modified FRPQ (draft SMS-FRPQ) which included 20 questions from the FRPQ verbatim, modified 8 questions, removed 1 question, and included 5 new questions. Three independent clinical SMS experts assessed the draft SMS-FRPQ and confirmed its content validity. Twelve caregivers of individuals with SMS who had participated in the original focus groups reviewed the draft SMS-FRPQ. Based on their feedback, we modified 13 questions (8 previously modified and 5 questions with new modifications). A second draft version was again reviewed by a new group of 19 SMS caregivers, and no further modifications were needed. The SMS-FRPQ sent for validation contained 21 questions, 7 verbatim from the original FRPQ, 8 modified from the original FRPQ, and 6 new questions. Each item had a 7-point response format with 0 = ‘never’ and 6 = ’always’. Two questions which asked specifically about verbal behaviors also had a “Not applicable” option to account for individuals who were non-verbal. These methods are in line with the MEASURE approach [26].

### SMS-FRPQ validation

Interested caregivers were directed to the online REDCap [22] portal and provided with a written overview of the research project. Questionnaires were filled out as proxy measures, as common for many disorders with impaired intellectual capacity. Subjects were asked to complete five questionnaires (described more below): demographics, SMS-FRPQ, HQ-CT, BPI-01, and the original FRPQ. We aimed to recruit at least 100 participants as this was felt to be both feasible in a rare disorder and was comparable to the original FRPQ sample size of 96 [34]. A total of 125 participants contributed data to the study, with 125 completing the SMS-FRPQ, 116 completing the HQ-CT, 108 completing the BPI, and 94 completing the original FRPQ. Since data were collected anonymously, we were unable to reach out to participants to complete missing questionnaires. There were no missing items from completed questionnaires.

### Measures

The measures/surveys collected in this study included the following:

- Demographics: Subjects were asked to provide their age, sex, race, ethnicity, zip code, and their relationship to individual with SMS. They were also asked to provide the following information about the individual they cared for: age, sex, race, ethnicity, living arrangement.
- Smith-Magenis Syndrome-Food-Related Problems Questionnaire (SMS-FRPQ): We developed the SMS-FRPQ, as described above. The following questions were reverse scored (utilizing final question numbers) for validation analysis: 3, 12, 15, 17. The final version (post-validation) is in Online Resource 1.
- Food-Related Problems Questionnaire (FRPQ): The FRPQ is a 16-item instrument designed to capture food-related behaviors and validated in the Prader-Willi syndrome population [34]. Original factor analysis was performed through expert consensus. The following questions were reverse scored: 3, 9, and 13.
- Hyperphagia Questionnaire for Clinical Trials (HQ-CT): The HQ-CT is a nine item instrument modified from the original 13-item Hyperphagia Questionnaire [16] for clinical trials [20]. The original Hyperphagia Questionnaire was validated in the Prader-Willi syndrome population but the HQ-CT was developed and tested in 17 subjects with PWS and did not undergo formal factor analysis or validation [20].
- Behavior Problems Inventory (BPI-01): The BPI-01 is a 52-item instrument designed to capture behaviors in individuals with cognitive and developmental delays on three subscales: self-injurious, stereotypic, and aggressive/destructive behaviors [33].

### Statistical analysis

#### Descriptive statistics

We calculated mean ± standard deviation (SD) for continuous variables (ages of the caregiver and individual with SMS), and frequency (N) plus percentage (%) for categorical variables, including binary variables race (White, Black, other), ethnicity (Hispanic vs. non-Hispanic), living arrangement (home with parent(s), group home, other), relationship to individual with SMS (mother, father, other), and geographical location (United States vs. other).

#### Internal consistency

We assessed internal consistency using Cronbach’s alpha (α) [13], with the calculation of 95% confidence intervals (CIs) for α calculated to include the variance of the covariance as recommended by Duhachek and Iacobucci [15]. A Cronbach’s alpha (α) coefficient > 0.7 is considered a good standard of reliability for research uses [25].

#### Exploratory factor analysis

The optimal number of factors was determined using parallel analysis [23], which uses Monte Carlo simulations (5,000 simulations per run) on randomly generated uncorrelated items, matched to our sample size and number of scale items, to produce eigenvalues for components or factors in the observed data that are adjusted for the sample error-induced inflation, and to formally test whether the number of factors was less than those that would be selected by chance. The number of components with adjusted eigenvalues greater than one, and that would not have been selected by chance based on the simulated data, were retained and fit to the observed data. Following Tabachnick and Fiddell [43], the initial solution was fit with an oblimin rotation, and the correlations among factors examined – where these generally reached r > = 0.3 and above, indicating an overlap in factor variance of > = 10%, the oblimin rotation was retained for the final solution. If this was not the case, a varimax rotation was selected, and chosen over no rotation where the majority of Thurston’s simplicity criteria [44] were met. The final model was specified according to the 0.40-0.30.40.30-0.20 rule [24], whereby (1) items were only retained which demonstrated factor loadings > = 0.40; and items only allowed to load into more than one factor where there were differences of > = 0.20 between their primary and alternative factor loadings.

The fit of the final model was assessed through the following fit indices: the root mean square error of approximation (RMESA [40, 41]) with RMSEA + 90% CI < 0.01 indicating an “excellent” fit, 0.01 < = RMSEA + 90% CI < 0.05 indicating a “mediocre” fit, and 0.05 = < RMSEA + 90% CI < 0.08 indicating an “adequate” fit [30], the comparative fit index (CFI [6]), and the Tucker–Lewis index (TLI [7]), with CLI > 0.9 and TLI > 0.9 both indicating a “good” fit [9].

#### Concurrent validity

Comparisons between questions asking similar constructs on the SMS-FRPQ (#6, 14, 5, 4) and HQ-CT (#1, 2, 6, 7 respectively) were analyzed in two ways. First, Spearman rank correlations were performed. Second, we compared responses on the SMS-FRPQ question between those whose answer on the HQ-CT question was 1–3 (less problematic) vs. 4–5 (more problematic) via multiple unpaired t-tests corrected for multiple comparisons. We also compared SMS-FRPQ questions # 6, 11, and 14 with the BPI-01 Aggression/Destructive subscale frequency score in a similar manner. First, we compared these questions with the subscale through Spearman rank correlation. Then, we compared responses on the SMS-FRPQ question between those whose Aggression/Destructive subscale score was < 11 or ≥ 11 via multiple unpaired t-tests corrected for multiple comparisons.

#### Age- and sex- effects

To test if differences existed in total or subscale scores in the SMS-FRPQ, we compared scores by age using three different age cutoffs: 4–10 y, 11–19 y, ≥ 20 y [16]. As noted above, each response was given a value 0–6 with questions 3 and 12 reverse scored, giving a possible range of 0–84, with higher numbers indicating more problematic behaviors. The “Takes Food” subscale was the sum of questions 2, 4, 7 and 13, giving a possible range of 0–24. The “Desire for Food” subscale was the sum of questions 1, 3, 5, 6, 9, 11, and 14, giving a possible range of 0–42. The “Satiety Impairment” subscale was the sum of questions 7, 8, 10, 12, and 13, giving a possible range of 0–30. We also compared scores by sex. Data were analyzed using two-way ANOVA and corrected for multiple comparisons using the Bonferroni correction.

## Results

To determine if existing tools adequately captured the food-related behaviors in individuals with SMS, we asked caregivers of individuals with SMS (*n =* 24) to complete the Food Related Problem Questionnaire (FRPQ) [34] and the Hyperphagia Questionnaire for Clinical Trials (HQ-CT) [12]. We then led discussions in focus groups about the ease of answering the questions and the applicability of these questionnaires for their child. Many caregivers struggled to know how to handle general questions about food seeking/taking because 1) their food was locked away and thus, the behavior was not seen often due to the external control and 2) their child only had disruptive behaviors with certain foods, not all foods. Caregivers also felt there were certain behaviors not addressed on the questionnaires, such as grazing and leaving food throughout the day. Since neither existing questionnaire was easily answerable by caregivers of individuals with SMS, we developed a modified FRPQ for the SMS population. After several iterations and expert review (see Fig. 1), we created a tailored questionnaire for the SMS population, the SMS-FRPQ (validation version) and asked caregivers from across the globe to complete it along with three other questionnaires (demographics, BPI-01, FRPQ).

Caregivers filling out the questionnaires were mostly female (87.2%), averaged 52 years old, and were predominantly non-Hispanic white (see Table 1). These participants represented the caregivers of individuals with SMS who were roughly half female (53.6%), averaged 21 years of age, with similar race/ethnicities to their caregiver, and predominately lived at home (80%) (see Table 1).

**Table 1:** Demographics of caregivers and individuals with SMS

Demographics of Caregivers of Individuals with SMS	*N =* 125
Female, %	87.2
Age, y	52.1 ± 11.2
Race, % (more than one race could be selected)	
White	96.8
Black	2.4
Other/no answer	4
Ethnicity, %	
Hispanic	2.4
Location, %	
US-based	63.2
Non-US based	25.6
Unknown	11.2
Relationship to Individual with SMS, %	
Mother	83.2
Father	12
Other	4.8

Demographics of Individuals with SMS	*N =* 125
Female, %	53.6
Age, y	21.0 ± 10.7
Race, % (more than one race could be selected)	
White	96.8
Black	4
Other/no answer	4.8
Ethnicity, %	
Hispanic	4
Living arrangement	
At home with one or both parents, %	80.0
Group home, %	12.8
Other relatives or arrangements	7.2

### Internal consistency

Our SMS-FRPQ had a coefficient of α = 0.87 [CI: 0.84–0.90], equivalent to the coefficient found in the original FRPQ publication of α = 0.87 (confidence intervals not published) [34] and similar to the FRPQ responses in our SMS population (α = 0.82, [0.76–0.86]). When considering individual FRPQ subscales, there was poor internal consistency for the “Impairment of Satiety” subscale of the original FRPQ in this population α = 0.22 [−0.11–0.37], while the internal consistence for the subscale “Composite Negative Behavior” was α = 0.76 [0.71–0.83], and “Preoccupation” was α = 0.65 [0.53–0.74], in our SMS cohort, indicating a benefit to revising the FRPQ for the SMS population.

### Factor analysis

**Fig. 2:**
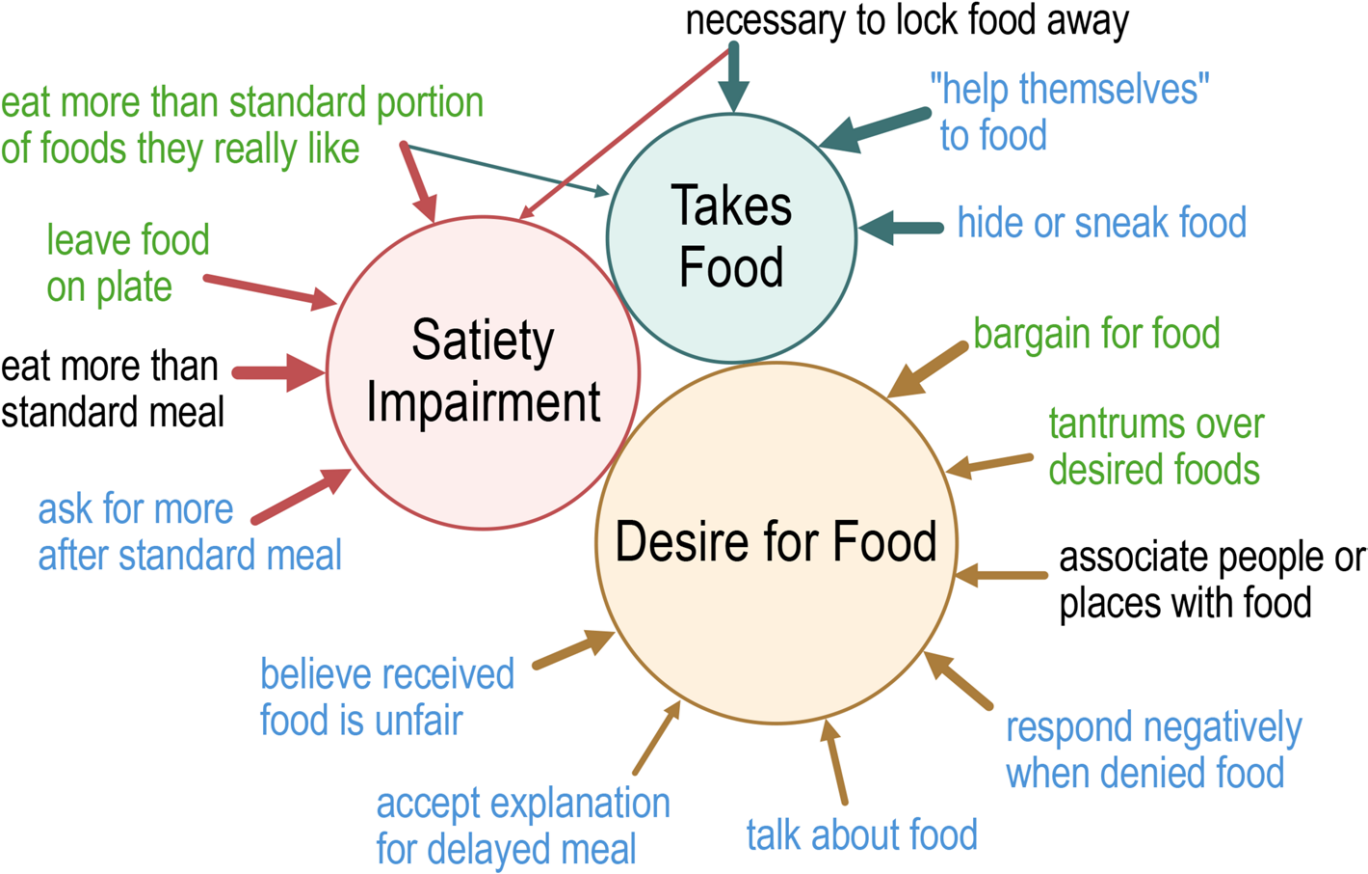
Visual representation of factor structure for the SMS-FRPQ. The SMS-FRPQ had three conceptual factors: Satiety Impairment, Takes Food, and Desire for Food. Questions loading onto each factor are shown in abbreviated form. Black text = questions unmodified from the original FRPQ, blue text = questions modified from the original FRPQ, green text = new questions not included in the original FRPQ. Width of arrows is proportional to the loading factor for each item

The original subscales of the FRPQ were created by consensus without formal factor analysis [34]. Thus, we performed an exploratory factor analysis for our SMS-FRPQ. A three-factor model with varimax rotation was selected, and 14 items retained, which fit indices suggested was close to a good fit to the observed data (CFI = 0.91, TLI = 0.88), and not significantly worse from an adequate fit (RMSEA = 0.09; CIs: 0.07–0.11.07.11). Retained items and their respective factor loadings in the final model are in Table 2 and represented in Fig. 2. In addition to a good overall data fit, the internal consistency of items loading onto each factor was good: Desire for Food, α = 0.80 [0.74–0.85]; Takes Food, α = 0.87 [0.83–0.90]; Satiety Impairment, α = 0.83 [0.77–0.87].

**Table 2:** Factor loadings for 3-factor solution for the SMS-FRPQ

	**Factors**		
**SMS-FRPQ Question Number**	**Desire for Food**	**Takes Food**	**Satiety Impairment**
1	0.684		
5	0.491		
6	0.677		
11	0.554		
9	0.591		
14	0.858		
3	0.362		
7		0.755	0.2
2		0.901	
4		0.815	
13		0.173	0.765
8			0.657
10			0.946
12			0.508

**Fig. 3:**
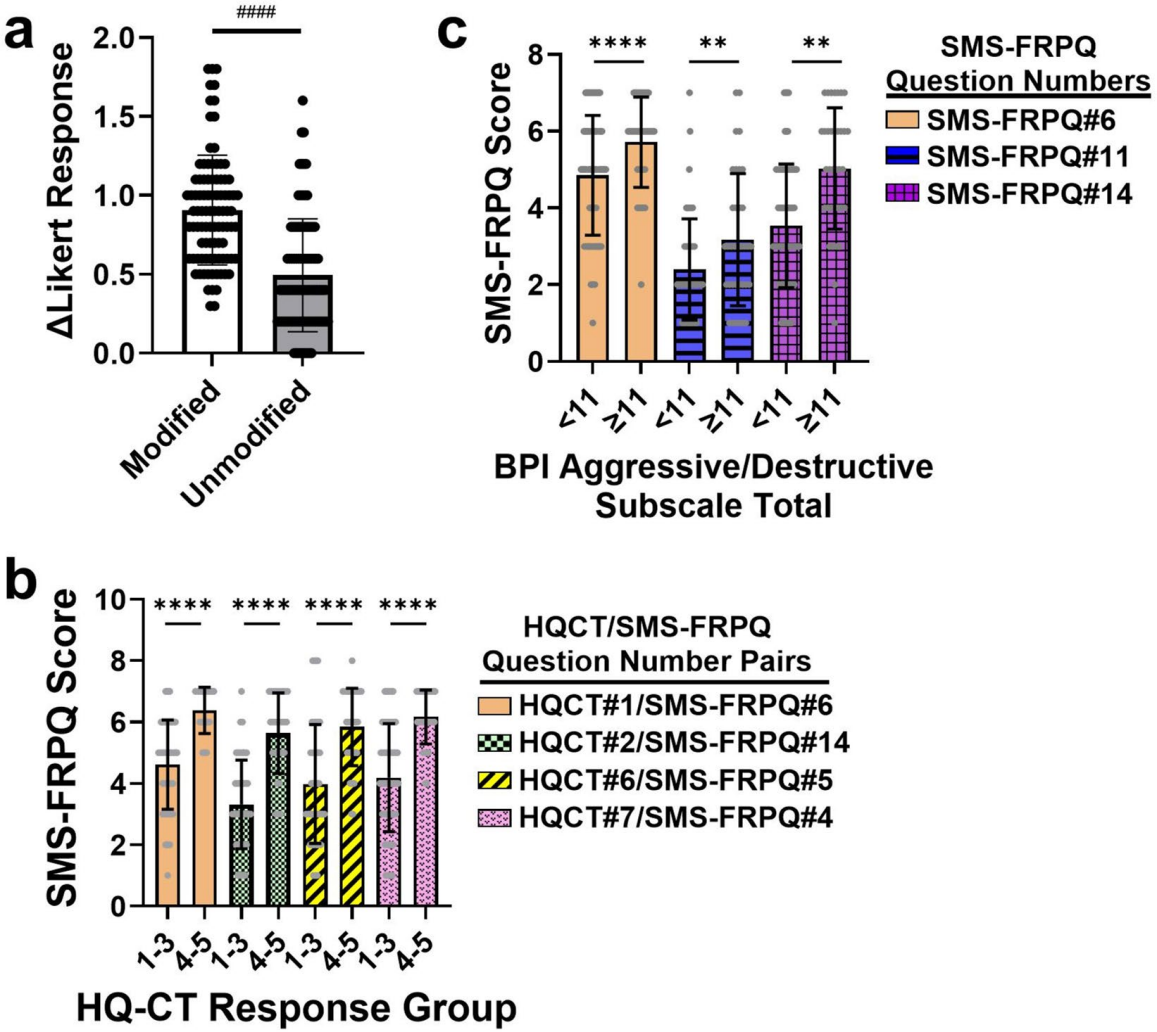
Comparison of Differences in Likert Scale responses on the SMS-FRPQ related to FRPQ, HQ-CT, and BPI questionnaires. **A** Differences in responses on SMS-FRPQ and FRPQ. Questions identical between the questionnaires are labeled “unmodified,” whereas those where small modifications were made are labeled “modified”. Differences between responses on the SMS-FRPQ and FRPQ for each type of question were averaged for each individual. **B** Concurrent validation of similar Items on SMS-FRPQ with HQ-CT. HQ-CT responses were grouped into those equal to Likert scale responses of 1–3 vs 4–5. Responses to the corresponding SMS-FRPQ question were then analyzed in each group. **C** Concurrent validation of negative behavior items on SMS-FRPQ with BPI aggressive/destructive subscale totals. BPI subscale totals were grouped as < 11 (corresponding to an average of less than monthly) and ≥ 11. Responses to SMS-FRPQ questions asking about negative reactions or behaviors were compared based on BPI aggressive/destructive subscale totals. ^####^*p <* 0.0001, paired t-test; **p < 0.01, unpaired t-test, corrected for multiple comparisons; ****p < 0.0001, unpaired t-test corrected for multiple comparisons

To determine if modifying questions from the original FRPQ resulted in changes in how caregivers answered questions, we compared average responses of caregivers between modified and unmodified questions, regardless of factor loading. Differences between each subject’s answers on modified (SMS-FRPQ questions 1, 2, 3, 4, 5, 6, 8, 17) vs. unmodified (SMS-FRPQ questions 7, 9, 10, 15, 16) questions were statistically significant (*P <* 0.0001 via paired t-test, Fig. 3a). At a question level, Spearman rank correlations of unmodified questions between the SMS-FRPQ and FRPQ ranged from ρ = 0.64–0.92 with 3/5 questions > 0.8 (Online Resource 2). However, the Spearman rank correlations of the eight modified questions ranged from ρ = 0.55–0.85 with only 1 of 8 > 0.8 [39, 42] (Online Resource 2).

### Concurrent validity

To assess concurrent validity (or how well the SMS-FRPQ correlates with a similar construct on a related instrument), we compared questions on the HQ-CT or BPI-01 to similar ones on the SMS-FRPQ. Correlations between similar questions on the HQ-CT and SMS-FRPQ were moderate to strong (ρ = 0.39–0.75; Online Resource 3). Answers to the HQ-CT are answered on a five-point Likert scale, with higher numbers indicating more problematic behavior. For the purposes of validation, we grouped responses from the HQ-CT questions into two groups, scores of 1–3 and scores of 4–5. We then compared responses on the corresponding SMS-FRPQ question between these groups (Fig. 3b; Online Resource 3). Responses to all four SMS-FRPQ questions showed significantly more frequent negative behaviors in those demonstrating more problematic behavior in the HQ-CT. For instance, significantly higher scores were reported for negative responses to denied food on the SMS-FRPQ (Question #6), between those reporting “very upset” or “extremely upset” compared to those reporting less reaction to being denied desired food on the HQ-CT (Question #1). Since some of the SMS-FRPQ items (Questions #6, 11, 14) related to possible aggressive behaviors, we analyzed if responses to these items corresponded to the aggressive/destructive behavior subscale on the Behavioral Problems Inventory-01 (BPI-01) (Fig. 3c, Online Resource 3). Positive correlations between the three SMS-FRPQ questions and the aggressive/destructive subscale of the BPI-01 were weak to moderate, ranging from 0.24–0.4. Responses to the BPI-01 are on a 4-point Likert scale ranging from 0 = Never to 4 = Hourly. Since the aggressive/destructive behavior subscale is 11 items, for the purposes of validation, we grouped subjects into those with behaviors averaging monthly or more occurrence (≥ 11) and those with very infrequent (< 11) subscale scores. We then compared responses on questions of the SMS-FRPQ. All three questions showed statistically higher frequency of negative behaviors in individuals who had clinically significant aggression/destruction (Fig. 3c).

### Age and sex

No significant differences were observed by age or sex in total or subscale SMS-FRPQ scores (Table 3).

**Table 3:** Comparison of SMS-FRPQ total and subscale scores by age and sex

	**Age, mean ± SD**			**Sex, mean ± SD**	
	**4-10y, *N =* 24**	**11-19y, *N =* 38**	** ≥ 20y, *N =* 63**	**Male, *N =* 58**	**Female, *N =* 67**
Total SMS-FRPQ Score^a^ (possible range 0–84)	43.4 ± 15.0	47.8 ± 14.2	46.8 ± 15.0	46.0 ± 16.1	46.8 ± 13.4
Satiety Impairment^b^ (possible range 0–30)	17.0 ± 6.5	19.1 ± 5.9	19.1 ± 4.3	16.0 ± 4.4	15.6 ± 3.6
Takes Food^c^ (possible range 0–24)	15.3 ± 6.1	18.9 ± 5.6	20.0 ± 6.3	17.4 ± 5.1	18.2 ± 4.1
Desire for Food^d^ (possible range 0–42)	29.6 ± 7.2	28.9 ± 7.0	26.8 ± 8.1	26.7 ± 8.2	30.6 ± 6.4

^a^Each response in SMS-FRPQ has a value 0–6 with higher scores indicating more problematic behavior. Total SMS-FRPQ score calculated by adding the sum of responses for questions 1–14

^b^Satiety Impairment subscale score calculated by the sum of responses to questions 7, 8, 10, 12, and 13

^c^Takes Food subscale calculated by the sum of questions 2, 4, 7 and 13

^d^Desire for Food subscale calculated by the sum of questions 1, 3, 5, 6, 9, 11, and 14

## Discussion

Our previous work demonstrated that individuals with SMS had specific and unique food related behaviors. No existing questionnaire fully captured these unique behaviors, and parents were conflicted when answering certain items on these questionnaires. Thus, we developed a questionnaire specifically for the SMS population. Our new questionnaire was developed with caregivers of individuals with SMS and underwent several rounds of editing to be clear and applicable to this population. Factor analysis showed that three factors emerged: Satiety Impairment, Takes Food, and Desire for Food. Our new, 14-item SMS-FRPQ had good internal consistency and concurrent validity. The SMS-FRPQ has a similar internal consistency using the Cronbach’s alpha coefficient to the original publication testing the FRPQ in the Prader-Willi syndrome population [34]. Together, these results show that the questions included in our new SMS-FRPQ measure a discrete set of interpretable latent constructs, with good psychometric properties, when assessed in the SMS population.

Our questionnaire development shown in Fig. 1 was in line with the established “MEASURE Approach” for instrument development [26], although modified since we did not develop a new tool from scratch but rather modified an existing tool. In Step 1, our purpose (M) was clear to develop an instrument that captured the breadth of food-related behaviors in the SMS population. Our empirical framework (E) was based on work in Step 2 testing the existing FRPQ (which utilized a consensus approach), HQ-CT, and our previous research [18]. We further developed our content areas (A) by discussing the number and types of items which needed to be added, based on our own experiences, the literature, and from caregivers themselves (also in Step 2). We then created new items (S) as a team and came to consensus internally in Step 3. We used expert review (U) from clinical content experts (Step 4) and caregivers (Step 6). We recruited participants (R) for a pilot version in Step 7 and a full validation version in Step 8. While we did not perform a full power calculation to determine sample size, we did set a goal sample size a priori based on previous literature. As a final step, we evaluated the validity and reliability (E) as described earlier.

Seven questions from the validation version of the SMS-FRPQ, including five from the original FRPQ, did not load onto the final SMS-FRPQ factor analysis. Two of these dealt with eating food not suitable for consumption. Working with caregivers, our qualitative work has demonstrated that individuals with SMS often do not display classic hyperphagic symptoms but instead show significant preoccupation and overconsumption of specific foods [18]. Thus, it is not surprising that so few caregivers noted these behaviors in their children. Given that 84–93% of responses were “Seldom” or less in these two questions, we did not include these in the non-factor loading “Additional Questions” section of the final SMS-FRPQ. Questions that did not load onto any of the 3 latent factors but still had clinical value were kept in the”Additional Questions” section of the SMS-FRPQ. Two of the five questions in this section were new questions requested by caregivers asking about eating unusual food (like condiments) or grazing. For all five “Additional Questions”, problematic behaviors rated as a 5 or higher occurred in over 25% of the tested population. Thus, these behaviors are important to assess, even if they fall outside of the three main constructs.

Setmelanotide is a medication targeting the melanocortin-4 receptor which has been approved to treat hyperphagia in 4 different forms of genetic obesity [45]. A previous trial with setmelanotide in the SMS population showed no difference in body weight measures over 12 weeks of treatment [29], despite a mean decrease in hunger measured by a two-item questionnaire. However, as noted above and in our previous publication [18], individuals with SMS do not have classic hyperphagia. The lack of appropriate measures for the SMS population may have masked the beneficial effects of that previous clinical trial due to an inability to understand how setmelanotide was positively impacting behavior without short-term impacts on weight. Had a more nuanced and accurate questionnaire been available for use, it may have been possible to understand the benefits and limitations of the medication to impact clinically-relevant behaviors in order to design a more effective trial in the future. The SMS-FRPQ provides an accurate measurement of these specific behavioral factors that might be useful in the course of a weight-related intervention or clinical trial. However, we acknowledge that longitudinal testing with this instrument has not been performed yet.

Improving obesity in SMS will likely require synergistic therapeutics that target distinct aspects of the syndrome, such as behavior, anxiety, and sleep, in addition to possible satiety-related therapeutics. Food-related behaviors may manifest themselves in response to not only hunger but also to several other contributing factors common to the SMS population such as abnormal circadian rhythms [11, 14, 27] and anxiety [2, 19]. Thus, it is likely that food-related behaviors may improve as sleep or anxiety are treated effectively. The SMS-FRPQ may also be useful as a secondary outcome in interventions trying to improve sleep or other behaviors (such as anxiety) in the SMS population.

This project was limited by the rarity of SMS. Standard methods of survey validation were not possible due to the low number of subjects and heterogeneity of the disorder. For instance, patient reported outcome measures included in the NIH Toolbox [21] were tested in ~ 500–3500 individuals [38]. We recognize that “good measures” are typically indicated by CFI or TLI > 0.95 or RMSEA < 0.06 with Cronbach α ≥ 0.7 [38]. While our analysis did not meet the stringent criteria for CFI, TLI, or RMSEA typically used for analysis involving much larger sample sizes, the SMS-FRPQ did have adequate fit, However, we relied heavily on input from the SMS population and their caregivers, as recommended in the literature [31, 35], to create a measure useful to the clinical and research populations. Additionally, we have used sample sizes that are similar to or better than other publications of similar questionnaires in rare populations [4, 34]. For instance, the FRPQ was originally validated in 93 individuals without formal reliability analysis and instead subscales created by expert consensus [34]. We also recognize that age of an individual typically has a significant impact on behaviors. Although we did not find any significant effects of age on the factor analysis, it is possible that a much larger sample size may reveal more differences in factor scores. Lastly, since we collected data anonymously, we could not perform a repeated measures analysis and so cannot draw conclusions about an improvement in fit on an individual level, but only at the group level.

We anticipate that future studies will benefit from the use of the SMS-FRPQ in clinical trials. This may require translation of the questionnaire into other languages as rare diseases often require international collaborations for enrollment. Future research would be helpful to understand the utility of this tool in children under 11 y and to determine if it could be used as a screening tool to identify food-related problems earlier in life. Studies utilizing the SMS-FRPQ as a repeated measure would be very helpful to determine the stability of answers over time from a validity standpoint and also to determine best practices around the frequency of utilizing the tool. Additional studies would also be useful to determine if use of the SMS-FRPQ clinically can aid targeted guidance or discussions with families to improve food-related behaviors.

## Supplementary Information

Supplementary Material 1.

## Data Availability

Data is provided within the manuscript or supplementary information files.
