# Supplementary material for: Investigating food-related behaviors in Smith-Magenis syndrome: tailoring a questionnaire for a rare disease
Source: J Rare Dis (Berlin). 2026 Apr 29;5(1):49. doi: 10.1007/s44162-026-00193-3 (PMC13124879; doi:10.1007/s44162-026-00193-3)
Supplement: Supplementary file 1 — Supplementary Material 1. [file 44162_2026_193_MOESM1_ESM.docx]

**Online Resource 1**

**Smith-Magenis Syndrome Food-Related Problems Questionnaire**

|  | Never | Almost Never | Seldom | Half of the Time | Usually | Almost Always | Always |
| --- | --- | --- | --- | --- | --- | --- | --- |
| 1. How often does the person believe the size or type of the food they receive is unfair? |  |  |  |  |  |  |  |
| 1. If given the opportunity (i.e. food is left unattended or unlocked how often would the person ‘help themselves’ to food which they should not have? |  |  |  |  |  |  |  |
| 1. How often is the person ever able to accept an explanation given to them if a meal is delayed? |  |  |  |  |  |  |  |
| 1. If given the opportunity (i.e. food is left unattended or unlocked), how often does the person ever hide or sneak food? |  |  |  |  |  |  |  |
| 1. How often does the person talk about food between meals? (Choose 'Not applicable' if the person doesn't speak) |  |  |  |  |  |  |  |
| 1. If the person was denied a food they really liked or wanted, how often would they respond negatively? |  |  |  |  |  |  |  |
| 1. Is it necessary to lock or hide desired food away to stop the person from taking food? |  |  |  |  |  |  |  |
| 1. After a standard size meal, how often will the person ask for more food? (Choose 'Not applicable' if the person doesn't speak) |  |  |  |  |  |  |  |
| 1. Does the person ever associate people and/or places with specific food items or occasions involving food? |  |  |  |  |  |  |  |
| 1. If given the opportunity, does the person ever eat more than a standard-sized meal? |  |  |  |  |  |  |  |
| 1. How often do tantrums or other disruptive behaviors happen when a meal or snack includes an item of food the person really likes or wants? |  |  |  |  |  |  |  |
| 1. Does the person ever leave food on their plate at the end of a meal? |  |  |  |  |  |  |  |
| 1. If given the opportunity, does the person ever eat more than a standard portion of a food they really like? |  |  |  |  |  |  |  |
| 1. How often does the person try to bargain or manipulate or throw a tantrum to get more foods they like or want? |  |  |  |  |  |  |  |
| **Additional Questions** |  |  |  |  |  |  |  |
| 1. If the person was tired, ill or upset, how often would this result in them going without food? |  |  |  |  |  |  |  |
| 1. If a meal includes an item of food the person does not like or is not expecting, how often would this result in behavioral difficulties? |  |  |  |  |  |  |  |
| 1. How frequently will the person willingly share favorite foods with others? |  |  |  |  |  |  |  |
| 1. If given the opportunity, how often does the person eat large quantities of unusual food items (e.g. ketchup, mayo, food coloring etc.)? |  |  |  |  |  |  |  |
| 1. If given the opportunity, how often would the person graze on food throughout the day (i.e. eat small amounts of food frequently throughout the day instead of large meals)? |  |  |  |  |  |  |  |

Scoring:

Questions 1-14 are scored 0-6 points, with “never” = 0 and “always” = 6. Questions 3 and 12 are scored in reverse (i.e. “never” = 6 and “always” = 0). Questions 15-19 do not get scored.

Scores for the following subscales can be added using scores from the listed questions:

Takes Food: 2, 4, 7, 13

Desire for Food: 1, 3, 5, 6, 9, 11, 14

Satiety Impairment: 7, 8, 10, 12, 13

**Online Resource 2**

Table 4. Spearman rank correlations for SMS-FRPQ questions unmodified from the FRPQ.

| **Questionnaire Item Pairs** | **Spearman rank correlation ρ** |
| --- | --- |
| ***Unmodified Questions*** | |
| SMS-FRPQ #7/FRPQ #7 | 0.85 |
| SMS-FRPQ #9/FRPQ #15 | 0.89 |
| SMS-FRPQ #10/FRPQ #16 | 0.83 |
| SMS-FRPQ #15/FRPQ #9 | 0.77 |
| SMS-FRPQ #16/FRPQ #11 | 0.64 |
| ***Modified Questions*** | |
| SMS-FRPQ #1/FRPQ #1 | 0.64 |
| SMS-FRPQ #2/FRPQ #2 | 0.75 |
| SMS-FRPQ #3/FRPQ #3 | 0.55 |
| SMS-FRPQ #4/FRPQ #4 | 0.78 |
| SMS-FRPQ #5/FRPQ #5 | 0.85 |
| SMS-FRPQ #6/FRPQ #6 | 0.74 |
| SMS-FRPQ #8/FRPQ #8 | 0.71 |
| SMS-FRPQ #17/FRPQ #13 | 0.76 |

**Online Resource 3 – Table 5 Comparisons of SMS-FRPQ with HQ-CT and BPI for Concurrent Validity**

| ***Comparisons of SMS-FRPQ with HQ-CT*** | | | | | | |
| --- | --- | --- | --- | --- | --- | --- |
|  |  |  | **Response on SMS-FRPQ for HQ-CT response group**  **Mean±SD** | |  |  |
| **SMS-FRPQ Question #** | **HQ-CT Question #** | **Spearman rank correlation ρ^a^** | **1-3** | **4-5** | **T(df)=F** | **Adjusted P Value** |
| Q6 - If the person was denied a food they really liked or wanted, how often would they respond negatively? | Q1 – During the past 2 weeks, how upset did the person generally become when denied a desired food? | 0.75 | 4.6±1.5 | 6.3±0.8 | t(92)=5.8 | <0.0001 |
| Q14 – How often does the person try to bargain or manipulate or throw a tantrum to get more foods they like or want? | Q2 – During the past 2 weeks, how often did the person try to bargain or manipulate to get more food at meals? | 0.72 | 3.3±1.4 | 5.6±1.3 | t(92)=7.3, | <0.0001 |
| Q5 – How often does the person talk about food between meals? | Q6 – During the past 2 weeks, outside of normal meal times, how much time did the person generally spend asking or talking about food? | 0.39 | 4.0±1.9 | 5.8±1.3 | t(92)=5.2 | <0.0001 |
| Q4 – If given the opportunity (i.e. food is lef unattended or unlocked), how often does the person ever hide or sneak food? | Q7 – During the past 2 weeks, how often did the person try to sneak or steal food (that you are aware of)? | 0.4 | 4.2±1.8 | 6.2±0.9 | t(92)=6.3 | <0.0001 |
| ***Comparisons of SMS-FRPQ with BPI-01 Aggression/Destructive Subscale Frequency*** | | | | | | |
| **SMS-FRPQ Q#** |  | **Spearman rank correlation ρ^b^** | **<11**  **Mean±SD** | **≥11**  **Mean±SD** | **T(df)=F** | **Adjusted P Value** |
| Q6 - If the person was denied a food they really liked or wanted, how often would they respond negatively? |  | 0.3 | 4.9±1.6 | 5.7±1.2 | t(106)=2.9 | <0.01 |
| Q11 - How often do tantrums or other disruptive behaviors happen when a meal or snack includes an item of food the person really likes or wants? |  | 0.24 | 2.4±1.3 | 3.2±1.7 | t(106)=2.6 | <0.01 |
| Q14 - How often does the person try to bargain or manipulate or throw a tantrum to get more foods they like or want? |  | 0.4 | 5.0±1.6 | 3.5±1.6 | t(106)=4.5 | <0.0001 |

^a^ Correlation between pairs of questions on SMS-FRPQ and HQ-CT; ^b^ Correlation between SMS-FRPQ question and BPI-01 aggression/destructive subscale frequency total score
